# Payers, Proof, and Public Trust: Lessons From Deep Brain Stimulation for Scaling Brain–Computer Interfaces

**DOI:** 10.1016/j.mcpdig.2026.100366

**Published:** 2026-04-25

**Authors:** Neilank Jha, Connor Liu, Aubrey Rogers, Andres Lozano

**Affiliations:** a Deep Brain BCI Corporation, Vaughan, Canada; b Department of Neurosurgery, Johns Hopkins University, Baltimore, MD; c Department of Neurosurgery, Albany Medical Center, Albany, NY; d Department of Neurosurgery, University of Toronto, Toronto, Ontario, Canada

Over the past 50 years, deep brain stimulation (DBS) has become one of the most effective neurosurgical therapies for neurologic disorders. The path from the first reported implants in the 1970s to its current acceptance as a standard-of-care in multiple medical conditions was not inevitable and required surgical and technological advances, regulatory support, and sustained industry investment.^1^ Since its first Food and Drug Administration (FDA) approval in 1997, DBS has improved the lives of more than 244,000 patients worldwide.^2^ Unfortunately, despite its proven efficacy, this only represents a small portion of at-need patients, and DBS remains underused in clinical practice. In the United States, only 1% to 2% of eligible patients with Parkinson disease (PD) or essential tremor undergo DBS annually.^3^ These gaps exist not because of scientific uncertainty but because of implementation barriers such as risk perception, latency of therapeutic benefit, and disparities in access.

As another generation of neurotechnology emerges, a similar story may be unfolding. Implantable brain–computer interfaces (BCIs) have progressed from laboratory prototypes to early clinical trials with promising results. Intracortical recording arrays and high-density subdural grids now enable communication and motor control at rates approaching functional utility for daily life. For example, real-time handwriting-to-text using intracortical signals achieved communication rates rivaling smartphone typing, whereas speech-decoding neuroprostheses restored sentence-level communication in people with anarthria.^4^^,^^5^ Human BCI’s roots trace to the late 1960s, where researchers first showed that electrical stimulation of the visual cortex in a blind volunteer can elicit light perception (Figure).^6^ It was not until the 1990s that long-term intracortical recording in a patient with locked-in syndrome showed that motor cortical ensemble activity could drive a computer cursor.^7^^,^^8^ The next major step was purposeful 3-dimensional action, and in 2012, 2 people with tetraplegia used a neural-driven robotic arm to reach, grasp, and self-feed.^9^

**Figure:**
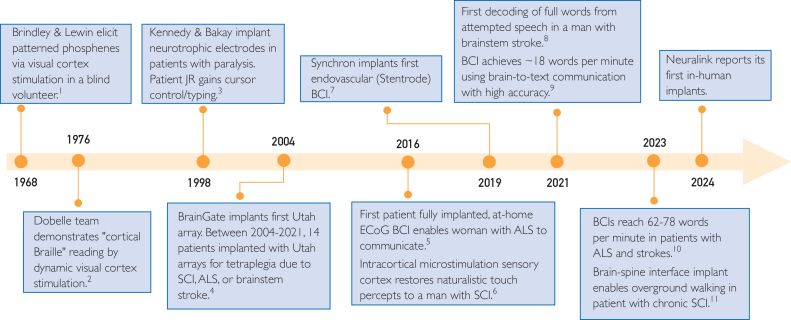
Timeline of human brain–computer interface (BCI) development. From left to right, chronological timeline of key events in the development of human BCIs. ALS, amyotrophic lateral sclerosis; EcoG, electrocorticography; SCI, spinal cord injury.

However, in-human BCI translation lagged for years owing to material limitations on signal stability, percutaneous hardware, power constraints, and immature decoders, which were not ready for home use or payer-relevant outcomes. In the last decade, converging technological advances have brought translational BCI technology to the forefront including fully implanted systems enabling independent home communication, bidirectional BCIs capable of restoring touch via intracortical somatosensory cortex stimulation, and algorithmic leaps to high-throughput communication via direct speech decoding.^5^^,^^10^^,^^11^ Together these milestones account for the present inflection in-human BCI capability and the transition from laboratory-bound prototypes toward durable, home-use neuroprostheses. To that effect, safety data from the BrainGate program, spanning more than a decade of intracortical array use, suggests acceptable rates of device or procedure-related adverse events in carefully selected patients, supporting continued feasibility studies.^12^ Therefore, DBS offers a roadmap for how BCIs might scale and where they might stall. In this study, we present 4 translational lessons that are particularly salient: (1) translating technology into patient-relevant value, (2) advancing clinical trials to include head-to-head comparison with standard-of-care, (3) aligning investment with clinical need, and (4) effectively communicating benefits and risks to gain public acceptance. By characterizing these features, we highlight the unique economic and cultural forces that will determine how BCI technologies unfold.

## Translating Technological Advancements to Patient-Relevant Value

Much of DBS’s early progress hinged on its ability to borrow from adjacent fields to improve patient outcomes. Stereotactic techniques were originally developed for ablative procedures and significantly reduced the morbidity of neurosurgical procedures.^13^ Additionally, the first implantable stimulators were an adaptation of cardiac pacemaker technology.^14^ Further device innovation has improved the therapeutic windows and usability of DBS, including directional leads to increase benefit and reduce side effects. Despite decades of advances, programming is still a time-consuming process to prevent an inappropriate dose of DBS, and further development is required in this area.^15^

Similarly, BCI has been able to improve its safety profile and advance its technology by borrowing from other fields. Synchron has developed an endovascularly deployed stent, decreasing the surgical invasiveness and tapping into a skillset without requiring training on a new procedural technique (Figure).^16^ Neuralink has adapted a robot to place the leads, decreasing surgical time and improving accuracy.^17^ Precision Neuroscience has developed an array that can be inserted through a microslit technique, eliminating the need for a full craniotomy and allowing for easy explantation (Table).^18^ Additionally, BCI needs to tie its advancements to tangible patient benefit, including more accurate communication, reliable home use with fewer maintenance visits, and easier explantation or revision. Without a clear vision for streamlining at-home use, patients and payers will stall in moving forward. This infrastructure includes not only devices such as assistive technology for at-home use but also personnel such as therapists, neuropsychologists, and information technology assistants.

**Table:** Summary of 21st Century Human Brain–Computer Interface Trials

Year	BCI indication	No. of patients	Type of BCI	Affiliation/location	Status
2004-2021	Tetraplegia: SCI, ALS, or brainstem stroke	14	Intracortical Utah array	BrainGate Consortium, USA	Complete
2014-2022	Cervical SCI	5	Intracortical array	University of Pittsburgh Medical Center, USA	Complete
2015-2016	ALS	1	Subdural electrode strip (Medtronic)	University Medical Center Utrecht, Netherlands	Complete
2019	Brainstem stroke	1	Subdural electrode strip (Blackrock Neurotech)	University of California San Francisco, USA	Complete
2019	ALS	1	Intracortical array (Blackrock Neurotech)	University of Tübingen, Germany	Complete
2019-2022	Tetraplegia	6	Endovascular Stentrode (Synchron)	University of Buffalo, USA	Complete
2023	ALS	1	Intracortical array	Stanford, USA	Complete
2023	Tetraplegia	1	Brain-spine interface (Medtronic)	NeruoX Institute, Switzerland	Complete
2024-2025	Tetraplegia: SCI or ALS	12	Robot-assisted intracortical array	Neuralink, USA	Underway
2025	ALS	3	Intracortical arrays	NeuCyber Neurotech, China	Underway

Compilation of in-human brain–computer interfaces described in published literature since 2000. Description includes date of trial, clinical indication for BCI implantation, number of patients, type of BCI array implanted, and trial affiliation.

ALS, amyotrophic lateral sclerosis; BCI, brain–computer interface; SCI, spinal cord injury.

Notably, in contrast to DBS where the subthalamic nucleus, globus pallidus internus, and ventral intermediate nucleus represent anatomically stable targets across patients, BCI relies on a diverse set of cortical targets. For example, motor and speech cortex exhibit variability in functional topography, requiring patient-specific cortical mapping to identify optimal recording sites and decoder training. This interindividual variability in cortical functional topography necessitates more personalized surgical planning and patient-specific decoder training. The DBS experience shows that technologies demanding specialized expertise diffuse slowly and concentrate in academic centers. Without deliberate efforts to standardize protocols and democratize skills, BCI threatens to remain a boutique therapy rather than achieving the population level reach its efficacy warrants.

### Moving Beyond Single-Arm Feasibility Toward Head-to-Head Trials

Although DBS was still in early development, the FDA introduced the Medical Device Amendments in 1976, which requires objective evidence of device safety and efficacy.^19^ This gave rise to the Unified Parkinson’s Disease Rating Scale, which provided an objective metric for reporting outcomes and allowed for large-scale trials to be conducted comparing DBS with the standard-of-care medical management at the time. Rigorous clinical trials have continued to refine patient selection, target selection, and timing of surgical intervention.

Brain–computer interface has accumulated an impressive portfolio of single-participant and small-cohort successes in communication (handwriting-to-text and speech decoding) and control (cursor and endovascular electrocorticography), with multiyear safety follow-up from BrainGate.^16^ To progress, the field now needs prospective, comparative studies measuring prespecified outcomes relevant to both patients and payers. Outcomes to be considered may include speed and accuracy in naturalistic conversation, daily device-use hours, caregiver burden, and quality-of-life gains. Durability end points should be defined to evaluate signal stability, reoperation rates, infection, and device replacement cycles. Trials should compare BCI-enabled workflows to the current standard-of-care assistive technology (eg, eye-tracking augmentative and alternative communication and switch scanning). Registries built on common data elements, analogous to movement-disorders registries that helped normalize DBS outcomes, can capture long-term safety and performance.

### Aligning Investment With Clinical Need in Setting of Long Adoption Cycle

Although DBS implants were initially proposed to treat patients with chronic pain, new FDA safety requirements began limiting their off-label use.^20^ Movement disorders, starting with essential tremor and dystonia, were identified as potential markets, and subsequently, PD provided a large enough patient pool to embolden investors. Today, 90,000 people are diagnosed with PD annually in the United States alone, and more than 10 million people in the world are currently living with PD.^21^ These reimbursement projections encouraged industry-led trials and sponsored studies to expand DBS indications.

Market reports project 12 billion in implant revenue for the BCI industry by 2045; however, the realizable market will be limited primarily by (a) candidacy funnels (clinical eligibility and psychosocial and caregiver support), (b) clinical capacity to implant and maintain systems, and (c) payer coverage.^22^ Even conservative estimates suggest a large pool including tens of thousands of people with high-cervical spinal cord injury, 17,000 with amyotrophic lateral sclerosis, and hundreds of thousands annually with poststroke aphasia or severe limb impairment.^23^, ^24^, ^25^, ^26^ If only a fraction ultimately meets criteria and consent to an invasive BCI, the near-term serviceable obtainable market is still attractive for health system–based business models. Private capital for BCI has been catalyzed by clinical milestones and adjacent neuromodulation successes such as DBS. However, those same lessons from DBS teach us that durable market formation will inevitably depend on coverage. Today, implantable BCIs remain investigational in the United States, and although Medicare covers routine costs of qualifying clinical trials, it does not fund the investigational device itself outside specific programs. To avoid the reimbursement uncertainties, sponsors should engage early with the Centers for Medicare & Medicaid Services and commercial payers, map trial end points to coverage criteria, and pursue pathways that facilitate postapproval evidence development (ie, Breakthrough Device designation). Indeed, Neuralink, Paradromics, and Cognixion, have all obtained Breakthrough Device designation for their BCI technology.

Deep brain simulation diffusion patterns offer a calibration point for BCI adoption curves. Despite randomized evidence and broad coverage, only a minority of eligible patients receive DBS, reflecting long penetration timelines. Mapping BCI scenarios to these historical patterns can inform enterprise valuations and health system capacity planning by tying revenue to realistic penetration and service needs. The DBS adoption curve in PD began with stepwise regulatory and coverage milestones: FDA approval for unilateral thalamic stimulation for tremor in 1997, expansion to bilateral subthalamic nucleus/globus pallidus internus for advanced PD in 2002, and national Medicare coverage in 2003. These decisions established both the clinical eligibility framework and the payer pathway that would govern diffusion over the next 2 decades.

To this end, Nationwide Inpatient Sample analyses show steady, decades-long growth in DBS procedures with PD, accounting for 67% of DBS discharges from 1993 to 2017.^3^ Thus, even with coverage in place, uptake accumulates gradually rather than in step functions. Larger perturbations can also occur. For example, hospitalizations for DBS fell sharply during the first coronavirus disease 2019 pandemic year, with national volumes dropping and monthly implants plunging by more than 90% in April 2020 relative to 2019, before partially rebounding.^27^

Despite growth, penetration among eligible patients remains low. Among 665,765 Medicare beneficiaries with PD in 2007 to 2009, only approximately 1% underwent DBS.^28^ Furthermore, persistent access disparities amplify this gap, such that from 2002 to 2018, Black patients with PD were 5 times less likely than White patients to undergo DBS, even as overall odds of placement increased over time.^29^ These patterns emphasize that randomized controlled trial–level efficacy and national coverage are necessary but not sufficient for broad, equitable adoption.

For BCI planning, the DBS experience implies 3 practical lessons for researchers and investors. First, model adoption as a slow, cumulative diffusion even under favorable regulatory and coverage conditions. This requires building operating plans that can withstand pandemic-level service interruptions and staffing constraints. Second, even with generous estimates the real-world BCI penetration will likely remain a fraction of epidemiologic eligibility for many years. Third, incorporate equity and geography into scenarios to include referral patterns, center availability, and social determinants that can meaningfully shape uptake. When capital markets or health systems value early BCI programs, tying revenue to realistic penetration, time-to-coverage, and lifecycle service needs rather than 1-time hardware counts will produce more credible ramps.

### Communicating Benefits While Addressing Cyber and Surgical Risks

If a transformative neurotechnology such as DBS, which is backed by decades of research, clear indications, and industry support, plateaued at just 1% to 2% utilization in patients, BCIs must gird for an even steeper climb given increased cyber security risks. Although most Americans oppose invasive implants as a means of elective enhancement, the majority support therapeutic brain chips that restore movement or treat disease.^30^ To this end, BCI manufacturers should align with FDA guidance on secure design. In 2025, the FDA finalized premarket cybersecurity guidance that expects secure architectures, software bills of materials, and patchability across the device lifecycle, all of which will need to be factored into overall costs, timelines, and postmarket operations.^31^ On surgical risk, BCI programs can adapt DBS playbooks: standardized preoperative evaluation, center-of-excellence criteria, perioperative infection-prevention strategies, and shared decision-making tools, which anchor expectations, including explant and reimplant probabilities over device life.

The pace of neuromodulation adoption may accelerate compared with previous decades because social media and open-access digital channels shorten knowledge-translation cycles among clinicians, patients, and payers. Although professional communities on X/Twitter and Free Open Access Medical Education routinely disseminate new evidence within days of publication, quality control and misinformation remain concerns.^32^ Indeed, digital networks can accelerate or thwart diffusion of medical innovation. For example, randomized trials in cardiothoracic surgery show that structured tweeting of new studies increases downstream attention and subsequent citations, a proxy for faster knowledge translation among clinicians.^33^ Conversely, amplification can misdirect practice: during coronavirus disease 2019, X/Twitter activity was associated with next-day changes in hydroxychloroquine prescribing ahead of negative randomized evidence.^34^ For BCI, these precedents argue for evidence-first outreach, transparent harms reporting, and active misinformation monitoring to harness the upside while minimizing downside risk.

### Looking to the Future

The near-term priorities for scaling BCI to its target patient population are clear. First, develop BCI technologies and infrastructures, which deliver durable function in the home with minimal daily setup. Second, launch comparative trials powered for patient-centered end points and payer-relevant value. Third, plan service models and supply chains that resemble other implantable neurostimulation fields, including training and technical support. Finally, pair scientific progress with policy work: develop consensus indications, registry infrastructure, and coverage frameworks, which ensure equitable access for spinal cord injury, amyotrophic lateral sclerosis, and stroke populations, which stand to benefit most.

In summary, BCI will not follow the DBS trajectory by accident. It will require the same combination of rigorous trials, relentless engineering, and pragmatic service design that ultimately made DBS a standard-of-care in PD. With that blueprint and with candid attention to safety, cybersecurity, and informed consent, BCI can scale from heroic case reports to reliable, reimbursed care for patients who need it most.

## Potential Competing Interests

Dr Lozano is a consultant for Abbott, Boston Scientific, Insightec, Medtronic, and Functional Neuromodulation and has implanted 2 patients with the Neuralink device. All other authors declare no competing interests.
